# Supplementary material for: Entropy production selects nonequilibrium states in multistable systems
Source: Sci Rep. 2017 Oct 31;7:14437. doi: 10.1038/s41598-017-14485-8 (PMC5663838; doi:10.1038/s41598-017-14485-8)
Supplement: Supplementary file 1 — Supplementary Information [file 41598_2017_14485_MOESM1_ESM.pdf]

Supporting Information to Entropy production selects nonequilibrium states in multistable systems

Robert G. Endres

Department of Life Sciences, Imperial College,

London SW7 2AZ, United Kingdom and

Centre for Integrative Systems Biology and Bioinformatics,

*Imperial College, London SW7 2AZ, United Kingdom**

*E-mail: r.endres@imperial.ac.uk

In this Supporting Information we derive equations used in the main text, and give additional explanations and results.

I. STEADY-STATE SOLUTION OF MASTER EQUATION FOR SCHLÖGL MODEL

The chemical reactions of the Schlögl model model with single species X are given by [1]

$$A \underset{k_{-1}}{\overset{k_{+1}}{\rightleftharpoons}} X \tag{S1}$$

$$3X \underset{k_{-2}}{\overset{k_{+2}}{\rightleftharpoons}} 2X + B, \tag{S2}$$

where A and B are clamped molecule numbers, driving the system out of equilibrium, and k_{+1}, k_{-1}, k_{+2}, and k_{-2} rate constants. The one-step chemical master equation for chemical species X can generally be written as

$$\frac{d}{dt}P(X;t) = \sum_{r=\pm1}^{\pm2} [W_r(X - \Delta X_r|X)P(X - \Delta X_r, t) - W_{-r}(X|X - \Delta X_r)P(X, t)] \tag{S3}$$

with the transition rates given by

$$W_{+1}(X|X+1) = k_{+1}A \tag{S4a}$$

$$W_{-1}(X+1|X) = k_{-1}(X+1) \tag{S4b}$$

$$W_{+2}(X|X-1) = k_{+2}X(X-1)(X-2)/\Omega^2 \tag{S4c}$$

$$W_{-2}(X-1|X) = k_{-2}B(X-1)(X-2)/\Omega, \tag{S4d}$$

for reaction volume Ω with $\Delta X_{\pm1} = \pm1$ and $\Delta X_{\pm2} = \mp1$ [2]. At steady state, the master equation can be solved exactly by iteration [3]. Introducing short notation

$$W^+(X) = W_{+1}(X|X+1) + W_{-2}(X|X+1) \tag{S5a}$$

$$W^-(X) = W_{-1}(X|X-1) + W_{+2}(X|X-1) \tag{S5b}$$

for the combined transition rates starting from X molecules, $dP/dt = 0$ produces

$$W^+(X-1)P(X-1) + W^-(X+1)P(X+1) - [W^+(X) + W^-(X)]P(X) = 0. \tag{S6}$$

Since this equation is valid for all X, the detailed balancing equation follows

$$W^+(X)P(X) = W^-(X+1)P(X+1). \tag{S7}$$

Hence, the 1D nonequilibrium problem can be mapped onto an effective equilibrium problem, which can be solved by iteration for the steady-state probability distribution and effective potential. Specifically, the steady-state probability distribution is

$$P(X) = P(0) \prod_{i=0}^{X-1} \frac{W^+(i)}{W^-(i+1)} = P(0) \exp \sum_{i=0}^{X-1} \ln \frac{W^+(i)}{W^-(i+1)}, \tag{S8}$$

and using concentrations instead of copy numbers, i.e. $x = 1/\Omega \sum_{X=0}^{\infty} X P(X)$ and scaling $p(x) = \Omega P(X)$, this leads to

$$p(x) = N(x) \exp[-\Omega \Phi(x)]. \tag{S9}$$

Here, the stochastic potential defined in the large-volume limit is given by

$$\Phi(x) = -\int_0^x dy \ln \left(\frac{\gamma^+(y)}{\gamma^-(y)} \right) \tag{S10}$$

with scaling $W^{\pm}(X) = \Omega \gamma^{\pm}(x)$ and the definitions $\gamma^+ = w_{+1} + w_{-2}$ and $\gamma^- = w_{-1} + w_{+2}$. The rates are now given by

$$w_{+1} = k_{+1} a \tag{S11a}$$

$$w_{-1} = k_{-1} x \tag{S11b}$$

$$w_{+2} = k_{+2} x^3 \tag{S11c}$$

$$w_{-2} = k_{-2} b x^2 \tag{S11d}$$

where $a = A/\Omega$ and $b = B/\Omega$ indicate concentrations. The remaining integral in Eq. S10 can be calculated analytically, producing [3, 4]

$$\begin{aligned} \Phi(x) &= x(\ln x - 1) + x \ln \left(\frac{k_{+2} x^2 + k_{-1}}{k_{-2} b x^2 + k_{+1} a} \right) \\ &+ 2\sqrt{\frac{k_{-1}}{k_{+2}}} \arctan \left(\sqrt{\frac{k_{+2}}{k_{-1}}} x \right) - 2\sqrt{\frac{k_{+1} a}{k_{-2} b}} \arctan \left(\sqrt{\frac{k_{-2} b}{k_{+1} a}} x \right) \end{aligned} \tag{S12}$$

(see Fig. S1, along with other potentials used in the main text). The volume-independent prefactor in Eq. S9 is given by

$$N(x) = \frac{k_{+2} x^2 + k_{-1}}{Z \sqrt{x} [x^2 + k_{+1} a / (k_{-2} b)]} \tag{S13}$$

with Z a normalization constant. Stochastic potential Φ has indeed minima at the stable steady states of the deterministic solution and a local maximum at the unstable deterministic steady state since

$$\frac{d\Phi}{dx} = \ln \left(\frac{\gamma^-}{\gamma^+} \right) \tag{S14}$$

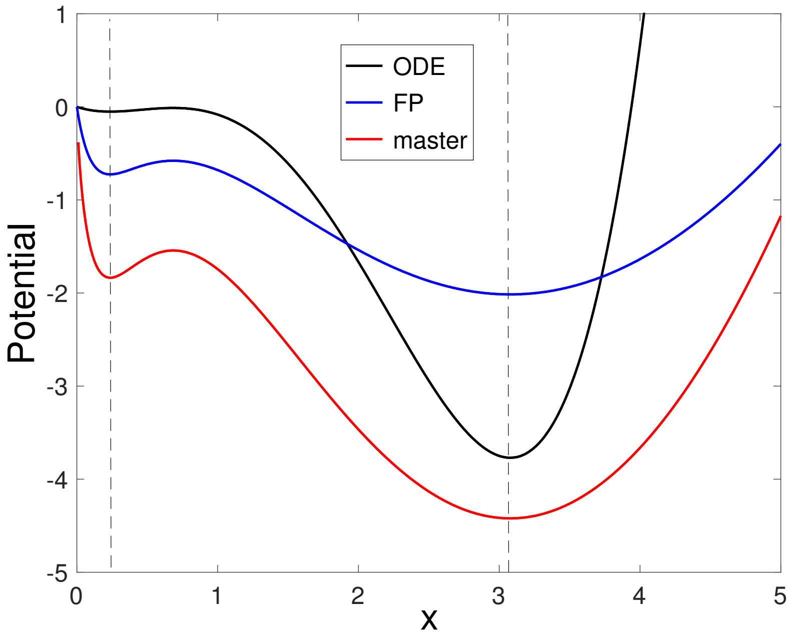

FIG. S1: **Effective potentials.** Comparison of effective potentials from ODE (black), Fokker-Planck (FP) equation (blue) taken from Eq. S30, and large-Ω limit of master equation (red) taken from Eq. 29 of the main text. The positions of the minima of the potentials coincide (vertical dashed lines), and the order of the minima is preserved. Parameters: $k_{+1}a = 0.5$, $k_{-1} = 3$, $k_{+2} = k_{-2} = 1$, $b = 4$, and $\Omega = 10$.

is zero for $\gamma^+ = \gamma^-$ and

$$\frac{d^2\Phi}{dx^2} = -\frac{\frac{d\gamma^+}{dx} - \frac{d\gamma^-}{dx}}{\gamma^+} = \frac{\Delta\gamma'}{\gamma^+} \tag{S15}$$

is > 0 (< 0) for stable (unstable) steady states with $\Delta\gamma' = d\gamma^-/dx - d\gamma^+/dx$. The sign of the second order derivative of Φ can easily be understood when considering standard linear stability analysis. Linearizing the ordinary differential equation of the Schlögl model (Eq. 14 in the main text) with $x = x_0 + \delta x$ around steady state x_0 produces

$$\frac{d(\delta x)}{dt} = -\Delta\gamma'(x_0)\delta x \tag{S16}$$

with perturbations decaying exponentially for $\Delta\gamma' > 0$.

II. TRANSITION RATES

Next, we consider the transition rates between the stable low (x_1^*) and high (x_2^*) steady states. Using a modified Fokker-Planck equation, known to correctly describe transport laws at large volume, the mean first-passage time is estimated from the splitting of the degenerate smallest eigenvalue [3, 5]. The transition rate for switching from the low to the high state via the unstable state (x_0) can then be calculated via

$$r^+_{1\to 2} = \frac{\gamma^+(x_1^*)\sqrt{-\Phi''(x_0^*)\Phi''(x_1^*)}}{2\pi\Omega} e^{-\Omega[\Phi(x_0^*)-\Phi(x_1^*)]} \quad \text{(S17a)}$$

$$= \sqrt{\frac{\gamma^+(x_1^*)}{\gamma^+(x_0^*)}} \frac{\sqrt{-\Delta\gamma'(x_0^*)\Delta\gamma'(x_1^*)}}{2\pi\Omega} e^{-\Omega[\Phi(x_0^*)-\Phi(x_1^*)]} \quad \text{(S17b)}$$

with short notation $\Phi'' = d^2\Phi/dx^2$. Similarly, the transition rate for switching from the high (x_2^*) to the low (x_1^*) state is given by

$$r^-_{2\to 1} = \frac{\gamma^+(x_2^*)\sqrt{-\Phi''(x_0^*)\Phi''(x_2^*)}}{2\pi\Omega} e^{-\Omega[\Phi(x_0^*)-\Phi(x_2^*)]} \quad \text{(S18a)}$$

$$= \sqrt{\frac{\gamma^+(x_2^*)}{\gamma^+(x_0^*)}} \frac{\sqrt{-\Delta\gamma'(x_0^*)\Delta\gamma'(x_2^*)}}{2\pi\Omega} e^{-\Omega[\Phi(x_0^*)-\Phi(x_2^*)]}. \quad \text{(S18b)}$$

Their ratio is given by the simpler expression

$$\frac{r^+}{r^-} = \sqrt{\frac{\Delta\gamma'(x_2^*)\gamma^+(x_1^*)}{\gamma^+(x_2^*)\Delta\gamma'(x_1^*)}} e^{-\Omega[\Phi(x_1^*)-\Phi(x_2^*)]}. \quad \text{(S19)}$$

Rates scale with system size and become exponentially small with increasing Ω. However, unlike the conventional Fokker-Planck equation, these rates depend on exact stochastic potential $\Phi(x)$.

III. DERIVING THE SCHLÖGL MODEL FROM GENERIC BISTABLE MODEL

The Schlögl model based on the ordinary differential equation

$$\frac{dx}{dt} = -k_{+2}x^3 + k_{-2}bx^2 - k_{-1}x + k_{+1}a \quad \text{(S20)}$$

can be derived from the generic bistable model

$$\frac{dx}{dt} = k_{+1}a + k_{-2}b\frac{x^2}{1 + \frac{k_{+2}}{k_{-2}b}x} - k_{-1}x \quad \text{(S21)}$$

often used in similar form in biology [6], ecology [7], and climate [8, 10] research. For example, in gene regulation x is the expression level of a protein (e.g. permease LacY in the bistable lactose utilization system [6]). In this case, Eq. S21 is interpreted as follows: the first term on the right-hand side represents basal expression, the second term is self-induced autoactivation by positive feedback, and the third term describes protein degradation. In another example from ecology [7], x represents the fraction of the lake surface covered by charophyte vegetation. Dramatic state shifts can occur through the sudden loss of transparency and vegetation observed in shallow lakes subject to human-induced eutrophication. Returning to Eq. S21, performing a Taylor expansion of the denominator of the second term for $k_{+2}x/(k_{-2}b) \ll 1$ to first order, we readily obtain the Schlögl model Eq. S20. However, in the Schlögl model, x can take any non-negative value, i.e. it is not restricted to small values.

IV. DERIVATION OF ENTROPY PRODUCTION FORMULA

In the main text we used the entropy production formula

$$\frac{ds_i}{dt} = k_B(R_+ - R_-)\log\left(\frac{R_+}{R_-}\right) \geq 0 \tag{S22}$$

with k_B the Boltzmann's contant (omitted in the main text). In Eq. S22, the first term represents the net flux while the second term describes the ratio of the affinity (or 'thermodynamic force') and temperature T given by the ratio of the chemical potential difference of the reaction and T, i.e. $\Delta\mu/T$. In equilibrium both the net flux and the chemical potential difference are zero. Here, we derive forumla Eq. S22 following [9], using the general chemical equation

$$\alpha A \;+\; \beta B \underset{k_-}{\overset{k_+}{\rightleftharpoons}} \gamma C \;+\; \delta D, \tag{S23}$$

where α, β, γ, and δ are stoichiometric coefficients, and A, B, C, and D are molecular species with concentrations a, b, c, and d. The rate constants for forward and backward rates are k_+ and k_-, respectively. Assuming the law of mass action, the forward and reverse rates are respectively given by $R_+ = k_+a^\alpha b^\beta$ and $R_- = k_-c^\gamma d^\delta$, so that the net flux (or the reaction velocity) is given by $R_+ - R_- = k_+a^\alpha b^\beta - k_-c^\gamma d^\delta$.

What is the chemical potential difference in terms of the reaction rates? In general, the chemical potential difference (difference in Gibbs free energy) is given by

$$\Delta\mu = \alpha\mu_A + \beta\mu_B - \gamma\mu_C - \delta\mu_D, \tag{S24}$$

where the chemical potential of some species X can be expressed as

$$\mu_X = \mu_X^0 + k_B T \ln(x), \tag{S25}$$

where μ_X^0 is the chemical potential at a reference condition and x is the concentration of X (where assume an ideal solution with activity coefficient equal one). With this in mind, the chemical potential difference Eq. S24 can be written as

$$\Delta\mu = \Delta\mu^0 \; + \; k_B T \ln\left(\frac{a^\alpha b^\beta}{c^\gamma d^\delta}\right). \tag{S26}$$

The first term on the right-hand side of Eq. S26 stems from the chemical potential difference at the reference condition $\Delta\mu^0 = k_B T \ln(K_{\text{eq}}) = k_B T \ln(c_0^\gamma d_0^\delta / a_0^\alpha b_0^\beta)$, where $K_{\text{eq}} = K_{\text{eq}}(T)$ is the equilibrium constant, only depending on temperature T but not on the chemical concentrations (or, more precisely, activities). The second term originates from the concentration-dependent log-terms of the chemical potentials. Upon further simplification, we finally obtain

$$\Delta\mu = k_B T \ln\left(\frac{k_f a^\alpha b^\beta}{k_r c^\gamma d^\delta}\right) = k_B T \ln\left(\frac{R_+}{R_-}\right), \tag{S27}$$

which proves Eq. S22.

V. INTERCHANGEABLE POTENTIALS

In Eqs. 24a-c of the main text, the gradients of the ODE potentials appear. Here, we derive the corresponding Fokker-Planck (FP) potential. The FP equation is another approximation of the master equation and constitutes a partial differential equation for the probability density function $p(a, b, x)$, containing contributions from the rate of change of the probability distribution due to currents (advection) and diffusion (noise) [11]. From the solution, the gradient of the potential would follow, e.g. $\Phi'_{\text{FP}}(a) = \partial\Phi_{\text{FP}}(a, b, x)/\partial a$. However, for sufficiently small noise at steady state, the three species A, B, and X can be

considered to fluctuate independently, and so we can derive the potential for each species separately. For instance, for species A the FP equation is (now without flux F)

$$\frac{\partial p(a,t)}{\partial t} = -\frac{\partial}{\partial a}\left[(w_{+1} - w_{-1})p(a,t) + \frac{\epsilon}{2}(g_a^*)^2\frac{\partial}{\partial a}p(a,t)\right]. \tag{S28}$$

where we assume that b and x are fixed at certain values (such as the steady-state values b^* and x^*, respectively). The steady-state solution is given by

$$p(a) \sim e^{-\Omega \int_0^a \Phi'_{\mathrm{FP}}(\tilde{a})\, d\tilde{a}}. \tag{S29}$$

The gradient of the potential for species A is

$$\Phi'_{\mathrm{FP}}(a) = \frac{w_{+1} - w_{-1}}{w_{+1}^* + w_{-1}^*} = \frac{\Phi'_{\mathrm{ODE}}(a)}{(g_a^*)^2}, \tag{S30}$$

which contrasts the exact large-Ω solution of the master equation $\Phi'(a) = \ln(w_{+1}/w_{-1})$ (in analogy to Eq. 12 of the main text). A similar potential can be obtained for species B. In Eq. 29 of the main text, we use first use Eq. S30 and subsequently replace the FP potential by the exact potential from the master equation for large Ω.

VI. PROOFS OF $\langle \dot{x}\Phi'_{\mathrm{ODE}}(x)\rangle = 0$ AND $\sqrt{\epsilon} g_a^* \langle \xi_a \Phi'_{\mathrm{ODE}}(a)\rangle = 1/2\epsilon(g_a^*)^2\langle \Phi''_{\mathrm{ODE}}(a)\rangle$

As outlined in the main text, we make the assumption that we can replace time averages by ensemble averages. In the main text we used $\langle \dot{x}\Phi'_{\mathrm{ODE}}(x)\rangle = 0$ for the 1D Schlögl model at steady state, where $\Phi'_{\mathrm{ODE}}(x)$ acts as a force from a conservative potential. While at steady state $\dot{x} = 0$ for the deterministic ordinary differential equation, $\dot{x}$ is not well defined in the Langevin equation due to the noise (jumps). Heuristically, we can argue for the ensemble average

$$\langle \dot{x}\Phi'_{\mathrm{ODE}}(x)\rangle = \left\langle \frac{dx}{dt}\frac{d\Phi_{\mathrm{ODE}}(x)}{dx}\right\rangle = \frac{d}{dt}\langle \Phi_{\mathrm{ODE}}(x)\rangle = 0, \tag{S31}$$

but here we will present a formal proof. Using Eq. 24a of the main text, we can proceed, now with an overall minus sign,

$$-\langle \dot{x}\Phi'_{\mathrm{ODE}}(x)\rangle = -\langle [-\Phi'_{\mathrm{ODE}}(x) + \sqrt{\epsilon} g_x^* \xi_x(t)]\Phi'_{\mathrm{ODE}}(x)\rangle \tag{S32a}$$

$$= \langle \Phi'_{\mathrm{ODE}}(x)^2\rangle - \sqrt{\epsilon} g_x^* \langle \xi_x(t)\Phi'_{\mathrm{ODE}}(x)\rangle \tag{S32b}$$

with $\xi_x(t)$ white noise multiplied by amplitude $\sqrt{\epsilon} g_x^*$ (i.e. constant effective temperature). Next, we need to calculate $-\sqrt{\epsilon} g_x^* \langle \xi_x(t) \Phi'_{\mathrm{ODE}}(x) \rangle = \langle \eta(t) G(x) \rangle$, where we redefined noise $\eta(t) = \sqrt{\epsilon} g_x^* \xi_x(t)$ and flux $G(x) = -\Phi'_{\mathrm{ODE}}(x)$. This calculation requires specification of the rule of discretization. Following [12] we Taylor expand the left-hand side of

$$G(x(t+\tau)) = G(x(t)) + G'[x(t+\tau) - x(t)]. \tag{S33}$$

Using Stratonovich (midpoint) discretization for the second term on the right-hand side of Eq. S33

$$\frac{x(t+\tau) - x(t)}{\tau} = \frac{G(x(t)) + \eta(t) + G(x(t+\tau)) + \eta(t+\tau)}{2} \tag{S34}$$

with $\langle \eta(t) \rangle = 0$ and $\langle \eta(t) \eta(t') \rangle = \epsilon (g_x^*)^2 \, \delta_{t,t'}$, we obtain for Eq. S33

$$G(x(t+\tau)) = G(x(t)) + \frac{\tau}{2} G'(x(t)) [G(x(t)) + \eta(t) + G(x(t+\tau)) + \eta(t+\tau)]. \tag{S35}$$

Hence, the quantity of interest from below Eq. S32b becomes

$$\begin{aligned} \langle \eta(t) G(x(t+\tau)) \rangle &= \langle \eta(t) G(x(t)) \rangle \\ &+ \frac{\tau}{2} \langle \eta(t) G'(x(t)) \, [G(x(t)) + \eta(t) + G(x(t+\tau)) + \eta(t+\tau)] \rangle \quad \text{(S36a)} \\ &= \frac{1}{2} \langle \eta(t)^2 \rangle \langle G'(x(t)) \rangle = \frac{\epsilon}{2} (g_x^*)^2 \langle G'(x(t)) \rangle. \quad \text{(S36b)} \end{aligned}$$

Note, since $G(x(t))$ and $\eta(t)$ are independent, $\langle \eta(t) G'(x(t)) G(x(t)) \rangle = \langle \eta(t) \rangle \langle G'(x(t)) G(x(t)) \rangle = 0$ (and similarly $\langle \eta(t) G'(x(t)) G(x(t+\tau)) \rangle = 0$) and $\langle \eta(t) G'(x(t)) \eta(t+\tau) \rangle = \langle \eta(t) \eta(t+\tau) \rangle \langle G'(x(t)) \rangle = 0$ due to white-noise property. Put into Eq. S32b this results in

$$-\langle \dot{x} \Phi'_{\mathrm{ODE}}(x) \rangle = \langle {\Phi'_{\mathrm{ODE}}(x)}^2 \rangle - 1/2 \epsilon (g_x^*)^2 \langle \Phi''_{\mathrm{ODE}}(x) \rangle = 0 \tag{S37}$$

since $\langle {\Phi'_{\mathrm{ODE}}(x)}^2 \rangle = \epsilon (g_x^*)^2 \langle \Phi''_{\mathrm{ODE}}(x) \rangle / 2$ due to an equipartition-type theorem for this effective equilibrium system when close to the potential energy minimum. To illustrate this, assume for simplicity a harmonic potential $\Phi_{\mathrm{ODE}}(x) = K x^2/2$. Now, we obtain relation $K \langle x^2 \rangle = T_{\mathrm{eff}}/2$, where $T_{\mathrm{eff}} = \epsilon (g_x^*)^2$ is an effective temperature. Hence, species X appears to effectively be at equilibrium without producing entropy.

In contrast to Eq. S37, when applying Eqs. S32b and S36b to molecular species A, we obtain instead

$$-\langle \dot{a} \Phi'_{\mathrm{ODE}}(a) \rangle = -F^2 + \langle {\Phi'_{\mathrm{ODE}}(a)}^2 \rangle - 1/2 \epsilon (g_a^*)^2 \langle \Phi''_{\mathrm{ODE}}(a) \rangle, \tag{S38}$$

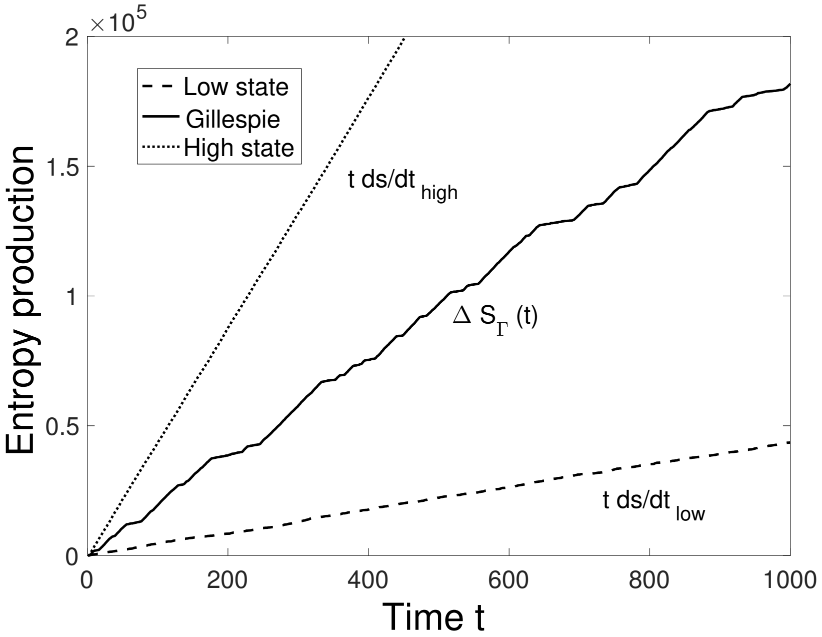

FIG. S2: **Entropy production from Gillespie trajectories.** $\Delta S_\Gamma(t)$ for a single trajectory of length t (solid line) and $t \cdot ds/dt$ from deterministic model (Eq. 15 of the main text) for low (dashed) and high (dotted) states (Fig. 3d) for $b = 4$ and $\Omega = 10$. Remaining parameters: $k_{+1}a = 0.5, k_{-1} = 3$, and $k_{+2} = k_{-2} = 1$.

which now contains imposed flux F. We further obtain $\sqrt{\epsilon} g_a^* \langle \xi_a \Phi'_{\text{ODE}}(a)\rangle = 1/2\epsilon (g_a^*)^2 \langle \Phi''_{\text{ODE}}(a)\rangle$, which we used in the main text. Now there is entropy production due to $\langle \dot{a} \Phi'_{\text{ODE}}(a)\rangle \neq 0$ from flux F, and similarly for species B.

VII. ENTROPY PRODUCTION DOES NOT DETERMINE PROBABILITY OF TRAJECTORY ALONE

Here we use simulations to show that trajectories are not solely selected thermodynamically based on maximal entropy production in line with the main-text result that the classical action needs to minimized as well. To investigate this, we use the entropy production defined by the log-likelihood ratio $\Delta S_\Gamma = \ln(P_\Gamma / P_{-\Gamma})$ with P_Γ and $P_{-\Gamma}$ the probabilities of the forward and backwards (time-reversed) trajectories Γ and $-\Gamma$, respectively. Specifically, we have [2]

$$\Delta S_\Gamma = \ln \frac{W_{r_1}(X_0|X_1) W_{r_2}(X_1|X_2) \ldots W_{r_n}(X_{n-1}|X_n)}{W_{-r_1}(X_1|X_0) W_{-r_2}(X_2|X_1) \ldots W_{-r_n}(X_n|X_{n-1})}, \tag{S39}$$

for n events during time t (see also Eq. 8 in the main text). Based on a trajectory, e.g. as obtained from exact Gillespie simulations for the Schlögl model, the numerator in the logarithm represents the product of all the transition rates, while the denominator contains the product of the transition rates in the time-reversed direction. As can be seen in Fig. S2, the slope fluctuates between low and high entropy production rates in line with main-text Fig. 2a,d. Ensemble averaging over many trajectories produces the average entropy production $\langle \Delta S_\Gamma \rangle$ [2]. If plotted for increasingly longer trajectory durations t, then the slope represents the average entropy production rate (up to a potential transient at the beginning if not initiated at steady state) [2, 13–15]. At equilibrium both numerator and denominator are equal and $\langle \Delta S_\Gamma \rangle$ vanishes as expected.

Coming back to the question at hand, we obtained trajectories by running Gillespie

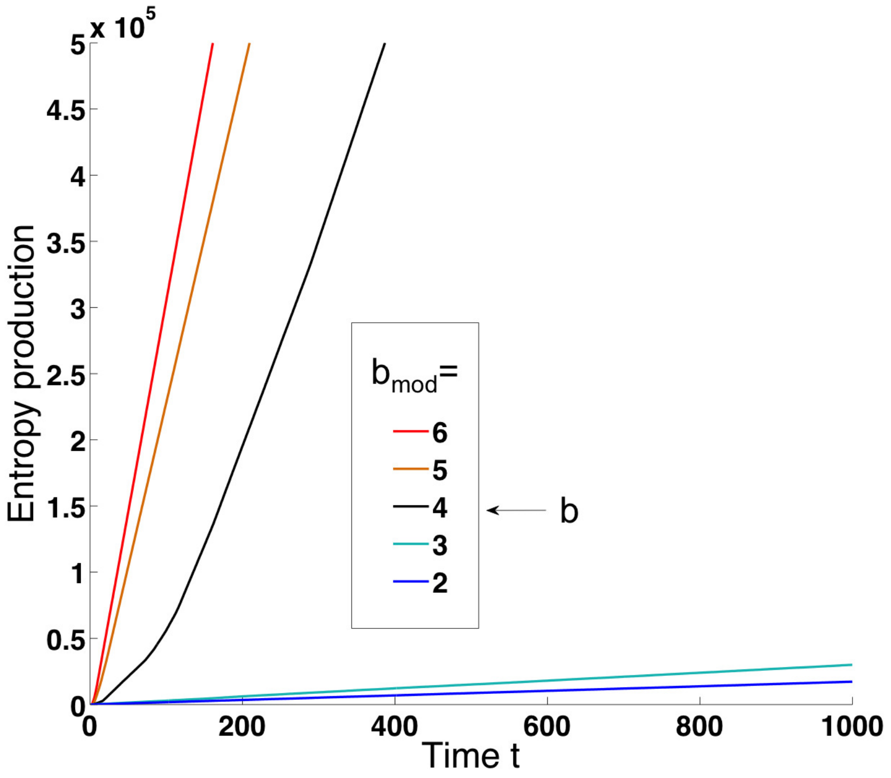

FIG. S3: **Test of maximum entropy production as sole criterion.** Ensemble-averaged entropy production $\langle \Delta S_\Gamma(t) \rangle$, where trajectories of duration t are created by the Gillespie algorithm for different b_{mod} values and the entropy production is evaluated by Eq. S39 for $b = 4$ in transition rate Eq. S4d (arrow). Parameters: $k_{+1}a = 0.5, k_{-1} = 3$, $k_{+2} = k_{-2} = 1$, and $\Omega = 60$.

simulations for different b_{mod} values and then evaluated $\langle \Delta S_\Gamma \rangle$ using a specific b value (here $b = 4$ inside the bistable regime). This way we are able to use perturbed trajectories, different from the correct trajectories that solve the master equation for a certain set of parameters. Fig. S3 shows that the ensemble-averaged entropy production $\langle \Delta S_\Gamma(t) \rangle$ is an increasing function of b_{mod}. In particular, the correct $b_{\mathrm{mod}} = b$ does not correspond to a maximum in entropy production. This can be understood by considering the log-likelihood ratio in Eq. S39. The entropy production is large whenever the probability of the forward trajectory is much larger than the probability of the backward trajectory. However, this statement is independent of how likely the forward trajectory is in the first place, i.e. whether it is a solution of the master equation. Hence, the maximum entropy production principle cannot be the sole determinant for selecting the most likely trajectory in line with the main text result.

[1] F. Schlögl, Chemical reaction models for non-equilibrium phase transitions. Z Physik **253**, 147-161 (1972).

[2] P. Gaspard, Fluctuation theorem for nonequilibrium reactions. J Chem Physics **120**, 8898-8905 (2004).

[3] P. Hanggi, H. Grabert, P. Talkner, H. Thomas, Bistable systems: master equation versus Fokker-Planck modelling. Phys Rev A **29**, 371-378 (1984)

[4] H. Ge and H. Qian, Thermodynamic limit of a nonequilibrium steady state: Maxwell-type construction for a bistable biochemical system. Phys Rev Lett **103**, 148103 (2009).

[5] M. Vellela and H. Qian, Stochastic dynamics and non-equilibrium thermodynamics of a bistable chemical system: the Schlögl model revisited. J Royal Soc Interfaces **6**, 925-940 (2009).

[6] E.M. Ozbudak, M. Thattai, H.N. Lim, B.I. Shraiman, A. van Oudenaarden, Multistability in the lactose utilization network of *Escherichia coli.* Nature **427**, 737740 (2004).

[7] M. Scheffer, S. Carpenter, J.A. Foley, C. Folke, B. Walker, Catastrophic shifts in ecosystems. Nature **413**, 591-6 (2001).

[8] M. Scheffer *et al.*, Early-warning signals for critical transitions. Nature **461**, 53-59 (2009).

[9] D. Kondepudi, I. Prigogine, Modern Thermodynamics: From Heat Engines to Dissipative

Structures (Second Edition, Wiley, 2015).

[10] A. Navarra, J. Tribbia, G. Conti The path integral formulation of climate dynamics. PLoS One **8**, e67022 (16pp) (2013).

[11] J. Hasty, J. Pradines, M. Dolnik, J.J. Collins, Noise-based switches and amplifiers for gene expression. Proc. Natl. Acad. Sci. USA **97**, 2075-2080 (2009).

[12] T. Tomé and M. J. De Oliveira, Stochastic mechanics of nonequilibrium systems. Braz. J. Phys. **27**, 525-532 (1997).

[13] J. Kurchan, Fluctuation theorem for stochastic dynamics. J. Phys. A: Math. Gen. **31**, 3719-3729 (1998).

[14] J.L. Lebowitz and D.J. Spohn, A Gallavotti-Cohen-type symmetry in the large deviation functional for stochastic dynamics. J. Stat. Phys. **95**, 333-343 (1999).

[15] U. Seifert, Stochastic thermodynamics, fluctuation theorems and molecular machines. Rep. Prog. Phys. **75**, 126001 (58 pp) (2012).
